# “Like a Dance”: Performing Good Care for Persons with Dementia Living in Institutions

**DOI:** 10.1155/2014/905972

**Published:** 2014-09-30

**Authors:** Kristin Mjelde Helleberg, Solveig Hauge

**Affiliations:** Institute of Health Sciences, Faculty of Health and Social Studies, Telemark University College, 3901 Porsgrunn, Norway

## Abstract

Dementia care is demanding, and health care workers can become emotionally exhausted and frustrated. Particularly, demanding aspects of dementia care include patient agitation and care-resistant behaviour. The aim of this study is to describe skilled staff's understanding of high-quality praxis in dementia care units in nursing homes. Eight nurses and care workers were individually interviewed, and a qualitative design was used. Participants were recruited from two nursing homes in two towns in eastern Norway. The data were analysed following the hermeneutic tradition inspired by Kvale. The analyses revealed three main findings describing good care: (a) to find: to identify the patient's personal characteristics, state, and needs, (b) to follow: to choose the right time and the tempo and to adapt to the patient's sensitivity, and (c) to lead: to be in the forefront and prepared and to change the patient's state. An overall interpretation of the findings is described by the metaphor of a dance between the patient and the caregiver.

## 1. Introduction

Employees working in institutional care for people with dementia have a challenging and demanding job due to agitation and other neuropsychiatric symptoms [1] and due to care-resistant behaviour (CRB) following the onset of dementia [2]. Agitation is described in terms of three agitation syndromes: aggressive behaviour, physically nonaggressive behaviour, and verbally agitated behaviour [3]. CRB differs from agitation because agitation may occur independent of a precipitating event, but CRB occurs in response to caregiver behaviour in the context of direct care [2, page 165]. A literature review based on 21 empirical studies concluded that personal care for the patients in nursing homes is the most frequent context in which aggressive behaviour occurs [4]. Nevertheless, both agitation and CRB challenge nursing care and nursing competence. The objective of this study is to explore and describe the characteristics of good care related to agitation and CRB among patients with severe dementia.

Several empirical studies have explored and described aspects of good nonpharmacological dementia care [5]. Cohen-Mansfield et al. [6, 7] underscore that there are staff-related barriers, environmental barriers, and system processes to reduce agitation. The importance of the staff is supported in several other studies. For instance, there are reasons to believe that employees' communication [8] and attitudes towards people with dementia and their knowledge about the disease and environmental treatment are important for preventing the occurrence of agitation [9, 10]. Bidewell and Chang [1] emphasised the importance of individual assessment and measures of meaningful activities or stimuli as well as a balance between social contact and solitude. Oppikofer and Geschwindner [11] analysed 433 documented cases with agitation and found that the most successful care interventions were avoiding noise, accompanying the person to the toilet, communication/validation, walking about/movement, and administering beverages. Kolanowski et al.'s [12] research indicates that activities performed twice daily reduce agitation, and Skovdahl et al. [10] showed that it is important for employees to have sufficient expertise and for both staff and patients to receive support.

In addition to studies specifically targeted at strategies to reduce agitation, there are also studies describing good dementia care in general. For instance, studies underscore that good care is characterised by respect, solidarity, and reciprocity [13, 14] and that good care involves individualised care [14, 15]. It is further indicated that facilitating flexibility and continuity are central to good care [16]. Sellevold et al. [13] describe good care as an employee's ability to sense what the patient expresses, to understand the patient's emotional and bodily expressions. Chung [17] found that care workers in nursing homes described good care based on its outcomes, exemplified by clean, comfortable, and happy residents, and as processes, exemplified by an affectionate, respectful, and patient attitude and mindset. Edvardsson and colleagues [18, 19] underscore the importance of the psychosocial climate for good dementia care in nursing home wards. They describe a supportive climate as a calm pace, a shared philosophy of care, possibilities of creating and maintaining social relationships, available and trustworthy staff, a willingness to serve, and a welcoming attitude [18]. To maintain a psychosocial climate, nurses and care workers need to be aware of their role in setting the emotional tone by sharing both the place and the moment with the patients [19].

The empirical findings described above are in line with a literature review by Traynor and colleagues [20], who define nursing competence in dementia care as understanding dementia, recognizing dementia, communicating effectively, assisting in daily living activities, promoting a positive environment, caring in an ethical and person-centred way, responding to the needs of family carers, facilitating preventive work, and promoting health. Overall, however, the findings of the studies presented above still leave us with few specific empirical descriptions of good care practices, as noted by Pulsford and Duxbury [21].

It is challenging to describe good care in terms of clinical competence. Nursing students, skilled nurses, and care workers can all benefit from contextual and more specific descriptions of good dementia care. Accordingly, the aim of this study was to explore how good care to patients with severe dementia in dementia care units in nursing homes is described by nurses and care workers.

### 1.1. Theoretical Perspectives

There are several theories describing good care, general nursing theories [22, 23], and more specific theories related to dementia care [24], all of which might shed light on the nature of good dementia care. Martinsen's theory [23] of caring can be interpreted as a description of the fundamentals of good care. She describes care as relational, moral, and practical. Relational care involves caring and concern for others. The moral dimension involves treating everyone as equal and with respect and requires the caregiver to understand the situation. The practical dimension means that care always depends on concrete and practical action. Another useful theoretical perspective is Benner's [22] description of what characterizes good care in the form of expert nursing competence. An expert has profound knowledge, acts intuitively, is flexible, and has a flow to his or her work. Expertise requires high analytical skills and a deep understanding of the situation based on common caring values [25].

These overarching theoretical descriptions are important for understanding what good care is. However, as Spichiger et al. [26] note, it is difficult to apply such context-independent descriptions to a specific clinical situation. There have been attempts to describe good care in the context of dementia care. McCormack and McCance [24] have developed a theory of person-centred care based on empirical studies [27]. The theory emphasises four elements:* prerequisites* focus on the attributes of the nurses,* the care environment* focuses on the context in which care is delivered,* person-centred processes* focus on delivering care through a range of activities, and* outcomes* include satisfaction with care. McCormack's theoretical perspectives are interesting, particularly due to the emphasis on the attributes of the nurse, the importance of the environment, and the characteristics of the process of caring and its outcome. All the theoretical perspectives indicate that nurses' and care workers' attitude, knowledge, and way of behaving towards persons with dementia are an important key to provide good dementia care.

## 2. Materials and Method

Data were collected through a qualitative design using in-depth individual interviews. This approach provided an opportunity to explore well-qualified and experienced nurses' and care workers' understanding of good care [28]. This methodological approach made it possible for the participants to reflect on and express what they considered the characteristics of good care.

### 2.1. Setting and Informants

The study was conducted at two nursing homes in eastern Norway. To meet the study's intent to describe good practice, we strategically selected one department in each nursing home that had a particularly good reputation due to its high quality of care. In Norway, we do not have a national ranking system. We therefore selected nursing homes that we knew to have a focus on the quality of care. One ward, with seven patients, was in a teaching nursing home where systematic efforts to improve the quality of care had been conducted since 2001. The other ward, with four patients, was in a nursing home that was specifically designed for patients with dementia and agitated behaviour and had a specialised staff. Both departments were designed for patients with advanced dementia. All patients had a private room with a bathroom, and they were encouraged to bring personal belongings to furnish their room. Both wards had a common living room and a kitchen unit available for the residents.

Experienced nurses and care workers were invited to participate in the study. Inclusion criteria were willingness to participate, status as a permanent employee in a more than 20% time position, and more than 3 years of experience in dementia care. Eight nurses and care workers (seven women and one man) volunteered to be interviewed. The characteristics of the participants are outlined in Table 1.

### 2.2. Interviews

Four interviews were conducted by the first author and four by the last author. The interviews were conducted in a quiet meeting room in the nursing home and were audiotaped. The interviews lasted from 50 to 75 minutes. During the interviews, the participants were asked to describe their background and their experience related to the main aim of this study. A thematic interview guide was developed. The interview guide was semistructured and included the following topics: description of situations in which good care was performed, how the interviewees assessed the patients' needs, perceptions of their own role in caring for people with dementia, experiences with the patients' agitation and strategies to prevent agitation, and their reactions to bad practice. The subjects were encouraged with follow-up questions such as, “Can you say more about…?”; “What did you do…?”; “What are you reflections about…?”; “How did you react…?”; and “Do I understand you right…?”

### 2.3. Data Analysis

The analysis of data from the interviews was inspired by the level of hermeneutic analysis described by Kvale and Brinkmann [29]. In short, the analytical process was developed by exploring the data in a circle from the whole to the part and from the part to the whole. In the first step, we listened to each interview and then read the transcribed text to obtain an overall and preliminary understanding of the data. In this process, we were struck by the participants' deep desire to understand and meet the patients' individual needs. In a joint discussion, we developed a preliminary understanding of the participants' descriptions of good care. In the second step, we read the material in a more detailed and systematic manner to identify categories that described good care. Texts from each of the categories were collected in separate documents and read through several times to gain an understanding of the nuances and details. In the third step, the categories were abstracted into three main themes: to find, to follow, and to lead. In this final process, we also developed an overall interpretation of good care, likened to a dance between the patient and caregiver.

### 2.4. Ethical Considerations

This project adhered to Norwegian legislation and was approved by the Norwegian Social Science Data Services (Project number 28475) and by the leaders of the two nursing homes. Nurses and care workers received written information about the study as well as oral information from the researchers. All participants signed an informed consent form before being included in the study. It was emphasised that the participants could withdraw their consent without any explanation.

### 2.5. Methodological Reflections

The study was conducted with only eight participants. This might be viewed as a small number that provided limited data. However, the participants were chosen purposively; they had good qualifications and extensive experience in dementia care. These qualifications were required to meet the study's intent to describe good practice. The researchers asked questions about the individuals' understanding and experience, and the participants were eager to share their experiences and reflected freely on their views of good care. Therefore, the eight interviews provided substantial data. It might be considered as a strength of the data that two researchers conducted and transcribed four interviews each.

## 3. Results 

The findings are presented in terms of three main themes. A detailed overview of the main themes and subthemes is presented in Table 2.

### 3.1. To Find

To find means to search the patient's personal characteristics, including his particular state of being and his overall needs. The participants expressed a common understanding of how important it was to gain as much information and knowledge about the patient as possible, including his life story. Emphasis was placed on looking for the individual patient, including his personality, character, behaviour, and habits: “We have seven patients, and we consider them completely different.”

The informants said that they attempted to see and understand the patient's condition here and now. They believed that it was necessary to understand the patient's condition to help the patient with daily activities. When they were attempting to identify the patient's condition, they were aware of the patient's communication, including body movement, rhythm, and language. They said that identifying the patient's current state was crucial. For example, in the morning, was the patient curled under the covers and not ready to get up, or was he sitting on the edge of the bed and ready to stand up? It was crucial to recognise and reflect on the patient's state for further interaction and to assess whether they would succeed in helping the patient at that time or if they had to wait. Knowledge of the patient and alertness about what the patient was attempting to express were the basis for exploring and understanding the patient's intentions: “I look at the facial expression—and looking how his eyes are—particularly if they are dark, that's important. Further, I look to see if he has a frown on his forehead, if the frown goes down to the nose or if it goes across the upper forehead. I also look for the state of his body—slouching (stooping) forward, or does he seem insecure? I try to read (his body language).”

Possessing and applying knowledge about basic needs, diseases, and symptoms were also vital for the participants in their quest to meet the patient's needs, particularly if the patient was troubled. They were looking for reasons for unease, such as pain, hunger, thirst, lack of sleep, or the need to go to the toilet. They looked for signs of illness, such as colds or urinary tract infections. In addition, they said that they were aware of psychosocial needs, such as worrying and longing for relatives. Identification of the patient's needs made the caregivers able to help the patients in most situations. The participants believed that this professional awareness enabled them to quickly reduce the patient's unrest and avoid resorting to medication to calm the patient.

### 3.2. To Follow

Based on the participant's understanding of the importance of identifying the patient's state, further implementation of good care was connected with the participant's eagerness to act in line with the patient's state by closely following the patient's situation. This concept was elaborated as timing, tempo and sensitivity.

The participants described the importance of choosing the right time for acting. If, for instance, it seemed impossible for the patient to get up in the morning or to brush his teeth, they said that it was no use to rush. When the patient refused or had dismissive body language, they left and tried again later. As one of them explained, “Yes, that's most likely the patient's mood. I see right away if I can…or I can't.” This constant awareness of the right timing also made them aware of the importance of acting when it actually was the right time for the patients. They were always prepared to intervene; they used opportunities, “golden moments,” to help the patient. The use of these opportunities depended on the staff's constant presence with the patient, either by sitting in the living room or by visiting the patients' rooms. Timing as a process of withdrawing, waiting, and trying again (one step forward and one back) was a common and general strategy described by the participants.

In addition to time, the participants underscored tempo as an important dimension of good care. Tempo involved the way they moved in the ward, talking to patients or colleagues, or the tempo while helping the patient to get dressed or eat. Other examples that the participants described involved ensuring that the bathroom was of a comfortable temperature so they did not have to bathe in a hurry, using quiet and relaxing sounds, and creating a feeling of silence and appropriate distance from fellow patients. They talked about how stress complicated interactions with the patient: “It says something, how I am, when I come into the patient's room...they notice quickly if I'm tight and stiff.” Maintaining a slow tempo could be particularly challenging if one of the other patients became agitated, for instance, by walking around restlessly. However, the staff's focus in such situations was to reduce their tempo by keeping peace, silence, and distance.

The participants further described following as an example of awareness of the patient's sensitivity to the staff's attitudes and actions. They found that patient “read” them, such as if they were stressed or worn out or had a bad day. The participants considered knowledge and awareness of themselves as professionals and their own attitudes and behaviours significant in preventing anxiety and acting out among patients: “It is therefore very important to work on oneself as a professional. There is much work to do with our own body language. You can try.” Interpreting the patient's signs and distinguishing between the patient's needs and their own needs were another crucial dimension of sensitivity. As an example of distinguishing between the patient's needs and their own needs, they mentioned that a quick and automatic action could be to draw the curtains in the morning and let the daylight into the room because that was what they needed, whereas the patient may see this as a brutal action that made further interaction difficult.

### 3.3. To Lead

To lead the patient involved helping the patient to live as healthy as possible despite his disease. The participants talked about their responsibility to be in the forefront to help the patients maintain order and control in their chaotic world. For instance, it was necessary to understand or know approximately when a particular patient usually needed to visit the toilet: “If a patient needs to go to the toilet, I try to lead. I try to show the way, maybe go ahead. So maybe the patient follows me, perhaps out of curiosity because they wonder what I will do.” Another example was the staff's focus on keeping the noise level as low as possible to prevent agitation. Doors into patients' rooms, common rooms, and the kitchen were closed when activities were happening there to strive for a peaceful atmosphere during meals.

The participants reported that considerable effort was put into different types of preparation, such as preparation before bathing, dressing, and meals. They specified things that was possible to prepare in advance. They prepared the patient for everything that would happen in different ways. For example, they might give the patient a hug when they turned him in bed or put a towel or rag in his hand to give him confidence. Turning the patient in bed could be a situation where the patient felt confusion. Another type of preparation was related to the person, such as being mentally present and focused on the individual patient.

The participants also talked about how important it was to change the patient's uneasy state. They did this in different ways, such as by helping the patient change focus by turning the conversation to something they knew was in the patient's interest. Patients were directed towards a good condition through praise and thanks for cooperation. They were encouraged and assured that “everything is fine” and that they had “plenty of time.” “I often say to the patient that we have time. It creates tranquility and safety for the patient that everything is okay because they so often get worried about anything that is or was.” This way of communicating was described as giving the patient “golden words” to bring the patient to a state of happiness, laughter, and enjoyment.

## 4. Discussion

The aim of this study was to explore how good care to patients with severe dementia in dementia care units in nursing homes is described by nurses and care workers. An overall interpretation of our findings (to find, to follow, and to lead) in this study is that good care can be likened to a dance between the patient and the caregiver. A dance involves activity, body movements, coordination, flexibility, endurance, and strength. A dance can express feelings and moods and create community; it can also have strict rules of procedures and room for personal interpretation and improvisation [30]. We believe that this concept captures what the nurses and care workers in our study told us about good dementia care.

Our participants' descriptions of good care for preventing agitation and CRB are similar to Benner's [22] general descriptions of good nursing. For example, our participants' eagerness to identify the state of the patient, to have sufficient knowledge about the disease, and to emphasise the single pertinent characteristic that distinguishes the patient as an individual fits well with Benner's [22] statement that an expert has profound knowledge. The importance of knowledge about dementia is also well documented in the existing research [9, 10] and in several literature reviews [1, 17, 21]. Furthermore, our findings (Table 2) described by the category “to follow” suggest Benner's [22] statements that an expert nurse acts intuitively and flexibly and has flow in his or her work. “To follow” was described by our participants as consisting of timing, tempo, and sensitivity. Such flexibility and willingness to follow the patient's state are supported by the results of several empirical studies [1, 13, 16, 17, 31].

An interesting part of the participants' descriptions of following the patient indicates that it was not only the staff who “read” the patient's condition but also the opposite: the patients were able to “read” the staff's situation and mood. Accordingly, sensitivity appears to be an important dimension, that is, relevant for both the patient and the carer. This approach reminds us of Martinsen's descriptions of good care as moral and relational [23]. The moral part is linked to a respectful and caring way of following the patient. This relationship was evident in the staff's sensitivity to the patients' needs. The importance of the carer's sensitivity is well documented by research [9, 10, 32]. However, the importance of patient sensitivity towards the carer is, to our knowledge, not very well documented in previous research.

Our study further emphasised that nurses and care workers said that they had to take the lead in the dance. This leadership rested on the two previous steps, to find and to follow. The nurses and care workers led the patient by being in charge of the situation and by being prepared and having a flow to their work [22]. Our participants understood the ultimate goal of leading to be the process of making the patient as confident and happy as possible [17]. The theme of leading can be interpreted as the key to good dementia care, as described by Martinsen [23]. It has been emphasised that good care encompasses solidarity and reciprocity [13, 14]. The nurses and care workers considered that they had to take the lead due to the patients' severe disease, but that their leadership had to be expressed in a sensitive and respectful manner.

Our overall interpretation was that good dementia care could be described in terms of the metaphor of a dance. Our impression is that the participants' descriptions of this “dance” were highly relational overall. The participants appeared to consider that good care depends first and foremost on the relationship between the carer and the patient. Such an approach to think and talk about good dementia care can rest on common knowledge about the disease of dementia, the theory of person-centred care [24], or empirical studies that emphasise the importance of relationships [18, 19] and communication [8]. Our participants were well educated and were, therefore, trained with a special emphasis on relational care for persons with dementia. However, it is interesting that our participants talked so little about physical activities as an important dimension of good care. This point is of particular interest in light of Kolanowski et al.'s [12] research, which indicates that activities performed twice daily can reduce agitation. One can wonder why practical nursing skills or activities were essentially absent from the statements about good care. Of course, this absence might have been related to the questions that we asked as researchers or to our exclusive use of a relational research method (interviewing). Additionally, the participants may have taken for granted that practical nursing skills and activities are part of everyday care and did not mention these topics for this reason.

After completing our analyses, we found an article that also used the metaphor of the dance. The article “The Dance of Caring Persons” described a relational model used to transform an organisation through caring values [25]. The model emphasises respect and expresses specific values, describing the key elements of care as knowledge, flexibility, patience, confidence, humility, and encouragement. Although that article focuses on the organisational level, it is interesting that our findings on a personal level appear so similar. Therefore, Pross and colleagues' study seems to support our findings [25]. However, in our understanding, to perform good care, nurses and care workers must show courage to involve themselves in a dance without knowing where it may lead them.

The results of this study contribute to detailed examples of how skilled and experienced nurses and care workers can facilitate good care. The participants' descriptions of good professional practice as finding, following, and leading can provide deeper insight into professional practice as an art. Further, using the metaphor of dance might help us to grasp the concept of good care in a more profound way. The study's results may help students, staff, and managers to analyse, evaluate, and adjust their own professional practice. However, there is a need for additional studies to further differentiate and describe the characteristics of good care.

## 5. Conclusion

The study shows that good care for people with dementia is characterised by the employees' eagerness to understand the patient's total situation, follow the patient's sensitivity by choosing the right time and tempo for intervention, and lead the patient to a calm and satisfied state. These characteristics correspond to current research and well-known theories about caring for people with dementia. Our study shows that good care can be likened to a dance. Further studies that examine the characteristics of good care in practice are necessary.

## Figures and Tables

**Table 1:** Characteristics of the informants participating in interviews.

Informant	Age	Education	Clinical experience in dementia care (years)
I	54	Care worker	30
II	42	Nurse (RN)	18
III	35	Care worker	13
IV	50	Nurse (RN)	4
V	49	Nurse with a master's degree (RN, MNS)	27
VI	39	Nurse with a master's degree (RN, MNS)	12
VII	50	Care worker, specialist in dementia care	31
VIII	43	Nurse specialist in dementia care (RN)	16

**Table 2:** Overview of the findings.

Like a dance		
To find	To follow	To lead
Searching for the patient's personal characteristics	Choosing the right time	Being in the forefront
Searching for the patient's state	Choosing the right tempo	Being prepared
Searching for the patient's needs	Adapting to the patient's sensitivity	Changing the patient's state
